# Unruly temporalities: Older queer women and non-binary people narrating later-life sexuality

**DOI:** 10.1080/08952841.2024.2428891

**Published:** 2024-12-09

**Authors:** Nika Looman, Ladan Rahbari, Katrien De Graeve

**Affiliations:** a University of Amsterdam, Amsterdam, The Netherlands; b Ghent University, Ghent, Belgium

**Keywords:** Queer temporalities, ageing, later-life sexuality, life courses

## Abstract

In this paper, we explore queer temporalities in relation to queer women and non-binary people’s sexuality later in life. Drawing on 30 interviews with 32 queer women and non-binary people aged 49-72 about sexuality and intimacy in later life, we highlight the participants’ stories about the instability and non-linearity of sexuality across the life course. First, we examine how our participants narrated later-life changes in their sexual subjectivity and how the assumption of compulsory (hetero)sexuality manifests in the participants’ stories about the unfolding of their sexual identities over the life course. We then analyze the compulsory non-sexuality imposed on women as they grow older. Finally, we explore the potential of reinterpreting sexuality in later life to destabilize pervasive normative notions of sexuality by analyzing the bodily changes the participants described. Rather than eradicating difference, such an analysis of later-life sexuality and queer temporality opens up the possibility of affirming changing desires and pleasures and acknowledging the body’s agency in shaping later-life sexuality.

## Introduction

Temporality refers to a social patterning of the experiences and understanding of time (Amin, 2014) and is inherently part of studies on life courses. The dominant understanding of life course revolves around life stages and milestones such as marriage, child-rearing, and generativity. Queer studies scholars identified these markers as normative and inherently linked to reproductive heterosexuality (Muñoz, 2009; Freeman, 2010). The temporal norms that impose life scripts have been theorized as ‘chrononormative’ (Freeman, 2010: 64) and linked to ageist discourse and its different impact on men and women (designated as ‘sexageism’, Carpenter & Higonnet, 1996, p. 142) regarding sex in later life. Chrononormative scripts result in older women being stereotyped as less interested in sex, less desirable, and less sexually attractive (Calasanti, 2005).

Drawing on the critique of chrononormativity, in this paper, we explore queer temporalities in relation to sexuality in later life. We build on the work of scholars such as Jack Halberstam (2005) and José Esteban Muñoz (2009), who have proposed a queer politics of time, pointing to the potential of queer temporality to disrupt normative temporal frames. Muñoz (2009) sees queerness as an ideal, a utopian future that proffers alternatives to the current heterosexual culture and the prevailing reproductive-based construction of the future. In Halberstam’s (2005) definition of queer time, a new temporality model emerges once one leaves the temporal framework of reproduction and longevity. Queer time or queerness itself is constituted by an alternative conception of time, which calls into question how dominant ideas of heteronormative time suggest that following the path of marriage, child-rearing, and generativity leads to maturity, happiness, and a link to a desirable future. These milestones orientate the life course in specific directions. As also discussed by Sara Ahmed (2006), this formulation of heterosexuality as an orientation is like a path or a line that directs us, but simultaneously is an effect of our work that affirms it.

Most analyses on queer time have typically focused on younger queer people and largely neglected the disruptive potential of queer later life. The few existing studies on queer later life (Sandberg, 2008, 2016; Wilkinson, 2020; Gallop, 2019) suggest that embracing the queer abject and failure enables a different way of thinking about ageing, normativity, and time. A turn towards old(er) age shows that ageing can too be identified as a queer endeavor, which has much to do with its embodied nature. In her theoretical essay, Linn Sandberg (2008) argues that queer theory and concern with old age and sexuality can be mutually enriching. Sandberg also argues that a turn to age can be useful for queer theory as it can potentially rethink sexualities, embodiment and notions of normalcy and sexuality, while performativity in queer theory and queer theory’s turn to embracing shame can be helpful for theorizing age differently. Preliminary research on non-normative genders and sexualities has illuminated the queer potential of time, such as Vanessa Fabbre’s (2014) work on older trans women and the work of Jones et al. (2023) on the intersection of bisexuality and ageing. These studies emphasize that awareness of time is an essential component of narrative identities, and by giving accounts of late-life change or continuing instability, they add that these narratives *queer* expectations about the stability of sexual and gender identities across the life course. However, sexual experiences and potential changes therein are largely left out of these studies. In other words, studies that aim to capture the production of queer temporalities by focusing on queer older women and non-binary people and ‘the changes, shapes, and fluidity of [their] bodies and desires’ (Sandberg, 2008: 119) are still missing.

In this study, we aim to make an empirical contribution to studies on life courses, temporality, and (queer) sexuality in later life. Using narrative analysis as a method, we centralize the stories of queer women and non-binary people in Belgium and the Netherlands, focusing on how they experience and narrate changes in their sexuality in later life. In the upcoming sections, we first present the existing literature on queer life course perspectives, queer temporality, and sexuality later in life. Subsequently, we provide an overview of our narrative methodology and describe our research methods. For our analysis, we draw on in-depth interviews with 30 queer women and non-binary individuals between the ages of 49 and 72. In our analysis, which is three-fold, we first address the participants’ narration of the unfolding of sexual identities over the life course. We continue with their stories of the impact of the discourse of compulsory non-sexuality on their (sex) lives, and end with how affirmative old(er) age enables a queer temporality that accounts for the later-life destabilization of these normative ideas of sex. We are ultimately interested in how the narrative work around queer temporalities of older queer women and non-binary people complicates current thinking about life courses and sexuality later in life.

### Sexageist chrononormativity: state-of-the-art

Life course approaches attempt to capture the flexibility of human life, viewing human life as a process shaped by various transitions, turning points, and thresholds in people’s life spans (Alwin, 2012). Heterosexual reproduction is central to chrononormative life courses, focusing on sex in adolescence and (early) adulthood, while sex in later life is often marked as off-limits as it is no longer about heterosexual reproduction (Freeman, 2010). Elizabeth Freeman (2010) elaborated on how the systems of sex and gender temporally order our lives through chronological paths and linear trajectories. She argues that the trajectory of sexuality unfolds differently for everyone, in a sequence of transitions that must be situated in their social and historical contexts. Even though the transitions and turning points significantly mark individuals’ life courses, different phases in life cannot be separated from one another. The cumulative aspect is also captured by Laura Carpenter (2010), who describes how sexual and social experiences at one point in a person’s life affect their beliefs later on. She argues that gender and sexuality are interwoven in the life course as an ‘ongoing biographical construction’ (157), pointing to how gender and sexuality are not fixed but rather unfold throughout chains of transitions that are embedded in historical contexts and individual events that potentially alter sexual trajectories in life (e.g., societal changes in sexual morality as a result of increasing secularization, ‘coming-out,’ or the onset of chronic illness).

Toni Calasanti (2005) points to how desirability and youthfulness are linked, resulting in (heterosexual) women no longer being viewed as potential sex partners once they enter midlife. Similarly, Paul Simpson (2021) uses the term ‘ageist erotophobia’ to refer to the failure or refusal to see older people as sexual beings, which has serious consequences for them. However, prevailing discourses of lifelong sexuality increasingly consider sex part of older women’s lives. Remaining sexually active is often understood as a way to resist growing old while ageing chronologically. But sexuality later in life is often framed within a paradigm of ‘positive’ or ‘active’ ageing, leaving its ageist underpinnings unquestioned. While ‘active ageing’ stands in opposition to the narrative that sees ageing as a ‘decline’ in terms of bodily and mental functioning, it remains normative in the sense that it accepts sex only when it stays within frameworks of heteronormativity, or more specifically: ‘heteronormative intimacy’ (Sandberg, 2016: 26). Linn Sandberg (2016: 26) has pointed out that it is not necessarily sex that is ‘celebrated as part of the good later life,’ but rather heterosexual intimacy: ‘a cluster of touch, sensuality, disclosure, feelings of love and commitment that hold particular significance to the heterosexual culture.’ Sex that does not adhere to chrono- and heteronormative expectations continues to be rejected or rendered invisible.

Although the normative temporality of sex desexualizes all older individuals, studies on older women and sexuality show that, on a subjective level, many older women still have or want sex. Contrary to dominant notions of sexuality later in life, the (predominantly heterosexual) women in their fifties and sixties in Margaret Rowntree’s (2015) research report that later life, particularly, can be a time for sexual exploration. Sandberg (2013) notes that older participants often describe later life as a phase of self-discovery or renewed or awakened interest in sex. Older (heterosexual) women are increasingly finding their way to digital dating platforms (Fileborn et al., 2015), and researchers also argue that older women may actually experience more confidence and freedom in their sexual relationships later in life (Watson et al., 2017) as well as increased assertiveness in sex and sexual pleasure (Miller, 2019). On the other hand, these researchers highlight obstacles faced by heterosexual women who seek sex later in life, which include a lack of success and a lack of truthful dating partners on digital dating platforms (Vandeweerd et al., 2016), a declining dating pool as men their age pursue younger women (Fileborn et al., 2015) and the perceived risk of losing independence when entering relationships with heterosexual men (Watson & Stelle, 2011). The reasons that older women may decide (not to) engage in new sexual relationships later in life, thus, are more complicated than just growing older.

While these studies show that older people both conform to and challenge chrononormative sexual life trajectories, they tend to construct dating and sex later in life in a narrow way that departs from heteronormative assumptions and privileges heterosexual intercourse as a defining sexual activity (Montemurro & Siefken, 2014). Much less attention has been paid to the ways in which queer women experience their sexuality as they get older, although the few studies that do exist highlight differences over time (Averett et al., 2012). Studies of queer and lesbian women’s sexuality, on, the other hand, have generally paid little attention to older women’s experiences. For example, Cohen and Byers (2014) show that lesbian women use different criteria for measuring their sexual satisfaction than heterosexual women, but how this affects older queer women is largely unknown in the academic literature, while as Jane Traies (2016) has noted, prevailing conceptualizations of sex are often too narrow (or heteronormative) to capture and understand the sex lives of older lesbians.

### A note on methodology

In this paper, we analyze the sexual stories of the participants of this research to study queer temporality in relation to the trajectories of sex in their life courses. We collected the stories via interviews conducted by Nika as part of their doctoral research on older queer women’s and non-binary people’s experiences of sexuality and intimacy in later life. Participants were invited to share their experiences with sex and intimacy later in life and how their relation to these topics changed throughout their life courses. We consider the stories as ‘active sense-making activities’ that ‘include explanations or theorisations of the past’ (Jackson & Scott, 2023: 479). As observed by Ken Plummer (1995) in his study of gay and lesbian coming out stories, sexual stories often follow a dominant pattern that begins in childhood and proceeds in a linear fashion. In narrating life courses in relation to sexuality, people draw on history, memory, and nostalgia to search for clues that help them make sense of their sexual lives. Our analysis starts from an interest in our participants’ sense-making through sexual stories. Searching for similarities and differences across the stories, and leaving room for inconsistencies, complexity and contradiction (Jackson & Scott, 2023), we aim to delve deeper into what they bring to the theorization of the temporality of sex throughout the life course, as well as to sexuality later in life.

The article is based on 32 in-depth interviews with 30 queer women and non-binary individuals between the ages of 49 and 72. Four people were interviewed twice, and some participants were interviewed together with their partners. The initial call for participants was aimed at lesbians, bisexual women, trans women, and queer women over the age of fifty, but it was later decided to include non-binary people as well. This decision was made after an initial round of interviews with people recruited through older LGBT+ people’s groups showed that several people had a more complex and temporally contingent understanding of their sexual and gender identity over the course of their lives than the categories we had been using. We realize that this description also had an impact on who did and did not feel addressed by our call. However, we tried to mitigate this issue by using different ways of recruiting participants (e.g. snowballing, participant observation in queer advocacy groups, and targeting specific people). By avoiding strict identity labels throughout the paper, we aim to reflect the complexity of the participants’ experiences and identifications.

The interviews were conducted by Nika between October 2020 and December 2022 and generally lasted two to four hours. Interviews took place in a location of the participant’s choosing, varying from community spaces or the terrace of a local queer organization to participants’ own homes or backyards. However, because of the several COVID-19-related lockdowns during the time of the research, some interviews took place via online video calls. Participants belonged to the white ethnic majority or were expats, and lived in either the Belgian regions of Flanders and Brussels or in the Netherlands. They were diverse in terms of socioeconomic status and living conditions. Interviews were conducted in a language that the participant and researcher had in common and in which the participants felt most comfortable, notably Dutch (25), English (6), and French (1). For the interview in French, Nika was assisted by an interpreter. As the majority of the interviews were in a language other than English, excerpts from the interviews in this paper are often translations. Before the interviews started, all participants were informed about the Later-in-Life Intimacies project and gave their informed consent. When written consent was difficult (e.g. because the interview took place remotely in a videocall), participants were sent all the necessary information prior to the interview and their oral consent was recorded. All names and locations in this paper have been pseudonymized. The paper is the product of the collaborative work of the three authors who have different ages (Katrien is in her early fifties, Ladan is almost forty, and Nika is in their late twenties) and have had different histories and non-linear gender and sexual identifications throughout their lives. The authors’ age differences and differential experiences with sexageism and, hence, relation to the data, contributed to the analysis and writing. The interviews are part of the Later-in-Life Intimacies project, which obtained ethical approval from the ERC ethical review committee (Ares (2020) 71491-07/01/2020) and from the ethical committee from the Faculty of Arts and Philosophy at Ghent University (08/12/2019).

## Findings

In the following, we present our analysis of the collected stories in three parts. We start with an analysis of how some of the participants discussed the end of their previous, long-term marriages. We pay attention to how the participants’ reinterpretation of their past sheds light on the ways in which compulsory heteronormativity and compulsory sexuality are embedded in the life course. In the second section, we show how sex and exploring new sexual identities later in life resist the desexualization of older people. Participants related ‘unruly’ temporalities to growing self-knowledge and self-esteem, but their stories also recount negative responses of people around them. In the last section, we apply the concept of affirmative old age to address both the anxiety around growing older described by the participants and the agency of the ageing body in their stories, which opens possibilities for rethinking sex and desire.

### Constructing queer pasts, constructing queer presents

The participants used terms as lesbian, bisexual, trans, queer, and/or non-binary to describe themselves. However, their life course trajectories and use of labels for gender and sexuality varied. Few found that identity labels such as ‘lesbian,’ ‘bisexual,’ or ‘trans’ were entirely accurate to describe themselves. For many, the idea of being a ‘woman’ had little meaning although they admitted that others often saw them as such. As this had a concrete impact on their position in society, they still found the category useful in mobilizing people to fight for social change. For some participants, labels had also been helpful in finding community, recognition, and a sense of self earlier in their lives, but were then discarded because they were perceived as too narrow to describe their sexual identities, experiences, and desires.

More than half of the thirty participants had been in long-term, monogamous marriages with men earlier in their lives. Some had married no later than the late 1960s, while the younger participants had married in the 1990s. Carine, who was in her seventies at the time of the interview, explained that marriage was what was generally expected in Catholic Belgium in the 1960s and 1970s. She said that it was both her family’s expectations as well as her desire to have children that made her want to marry a man. However, she recalled having pleasurable sexual relationships with women before her marriage, which disrupts the heteronormative temporality of her sexual history. Jo’s story shows similarities even though she was almost twenty years younger than Carine and had married a man in the 1990s. She said about the sex with women before her marriage: ‘It was very exciting, but also something that I thought would go away.’ Jo now presented this thought as naïve, as her desire for women did not disappear. She explained this naïveté with her childhood in a devout Catholic family where a lesbian relationship was out of the question. Life courses are always constructed in particular historical and cultural moments (Hammack & Cohler, 2009), and so were Carine and Jo’s. Both participants foregrounded historical and cultural contexts to explain changes and life choices, and they subsequently created a sense of continuity in their sense of self. Plummer (1995) argues that sexual stories are ‘a constant readjustment,’ depending on the time and place in which they are told. Contemporary narrative templates for queer sexual stories typically require both linearity and a story of gradual liberation of a ‘true self’. Carine and Jo described both by highlighting particular events and feelings that fit this consistent story, while explaining elements that escape linear time through naïveté or societal pressure.

Martha, who was almost 50 at the time of the interview, had married her ex-husband in the early 1990s. She mentioned her affluent family’s expectations as a motivation to marry a man. She described entering this relationship as follows:

I didn’t understand why I wouldn’t fall in love with any of the men I met […], so when I was 24 or 25 years old, I met someone at work and he was head over heels in love with me, so I thought, okay, why not? […] I was in that relationship, had two children, we married, but having sex, cuddling… it did not interest me at all. For a while I even thought I was asexual.

Like the stories of Carine and Jo, Martha’s story also stands in tension with the Western, late modern romantic ideal of marriage, which is based on mutual sexual and emotional attraction, and solely the result of individuals’ choice. However, in contrast to Carine and Jo, Martha emphasized that she had not been ‘aware’ of her sexual desire towards women at the time. Therefore, Martha had not experienced her choice to marry a man as suppressing her lesbian sexual desires, but rather explained this choice through the lack of access to alternative sexual imageries that had made her go with the flow of heteronormative expectations. This trajectory was the only one available or the only one presented to her as a possibility at the time. The sexuality that she presents in her story is in line with the ‘sexuality as orientation’ model as formulated by Sara Ahmed (2006: 547). According to this model, ‘one’s background affects what comes into view’ and ‘[s]ome things are relegated to the background to sustain a certain direction.’ Sexuality as orientation is then about ‘a direction (taken) towards objects and others’ (557), which affects patterns of relating to others and makes both straightness and queerness about continuous becoming, not being.

However, Martha described how ‘the puzzle pieces’ of her sexuality came together later in life when she fell in love with her current partner, Dominique. She described how from then on she began to reinterpret her previous crushes on female teachers in high school as an indicator of her lesbian sexual orientation. Narrating her trajectory in this way, she drew on a popular storyline of queer sexualities that require queer people to see their sexuality in terms of identity and find “clues” of queer sexuality in one’s past. These clues reconfigured her sexuality as consistent, despite Martha’s inability to interpret her feelings towards women as sexual desire earlier in her life. In Martha’s story, not only did her past reshape her present, but her present also shaped her history (as she was only able to interpret the “clues” in her past after she identified her sexuality as queer). Thus, we follow Plummer (1995) in recognizing that present and past cannot be separated or placed in a linear (albeit not straight) trajectory.

Martha’s doubts about her sexuality earlier in life illustrate that ‘compulsory heterosexuality’ (Rich, 1980) prescribes a linear path that leaves only one option for those not interested in heteronormative sex, and that is asexuality. Scholars in asexuality studies (see Chen, 2020; Przybylo, 2019) have built upon Rich’s concept to argue that compulsory sexuality shapes every stage in life. From a normative life course perspective, the obligation to be sexual manifests itself in the expectation that everyone becomes sexual at a certain age (in their teens) and then stops being sexual (around menopause for women or even earlier). Participants who had found clues about their sexual identity earlier in their lives often used the feeling of relief or reassurance to describe how they felt when they realized that they were not asexual, but simply not attracted to men. In those stories, asexuality was not an affirmative sexual identity, but something that is ‘wrong’ with the person who has no interest in heterosexual sex and intimacy. Martha’s confusion about her sexuality disappeared when she fell in love with her current partner a couple of years before the interview. She described how she began to enjoy sex, implying that sexual pleasure and a sexual self-awareness only emerged later in her life. However, the hegemonic discourse on later life portrays this phase of life as one in which sexual desire is stereotypically expected to decline and when women are supposedly less and less sexually desirable. Martha’s story therefore destabilizes not only heteronormative life courses, but also the narratives that desexualize women later in life.

### Compulsory non-sexuality and (queer) temporality

Other participants also resisted the contemporary hegemonic discourse that portrays women as less sexually desirable and less interested in sex later in life. Vera, who was in her fifties at the time of our interviews, explained how her desire to have sex had actually increased rather than decreased over the years, particularly after she had entered menopause. She explained that her second long-term marriage to a man had resulted in a divorce, because of discrepancies in their sexual desire. Vera emphasized that her ex-partner’s interest in sex had declined over the years, and the differences in desire had been a prominent point of disagreement before they separated. Vera said,

He said, ‘that’s what happens when you get older’ and I was like, ‘no, that doesn’t happen when you get older!’ He kind of tried to excuse himself and then we had so many talks about this and he never really wanted to acknowledge that what was going on with him was problematic for me, because my libido kept rising. At a certain point we were [almost 20 years] together, and so I had to come to terms with the fact that if I stay in this relationship, it’ll be in a sexless relationship. But [the lack of] sex itself was not the worst, it was the lack of intimacy.

Vera explained that it was important for her to express and explore her need for sex, despite ageist stereotypes and the desexualization of women later in life. She described her need to explore sexual fantasies as something that had been ‘simmering’ for a long time. ‘Simmering’ fantasies imply that Vera’s curiosity for non-normative sex had already been present when she was ‘still’ following a supposedly (hetero)normative path in life. A queer temporality, one that undoes the heteronormative life course, thus already coexisted or intertwined with a more heteronormative one.

Like other participants that had divorced later in life, Vera described that she was initially hesitant to leave her long-term marriage. The hesitation they felt was often described by the participants as a result of societal expectations imposed on women later in life, and more particularly, the hegemonic discourse that desexualizes older women. Even if their long-term relationships were no longer (sexually) satisfying, participants were reluctant to seek new sexual and/or romantic relationships because they feared being perceived as (sexually) unattractive by future potential partners because of their age. The participants’ hesitation demonstrates women’s awareness of the pervasive sexageism that structures the dating market. But their stories also reflect – as has also been noted in research on ageism (see Moore & Reynolds, 2016) – their internalized insecurities. Some participants shared anxieties about dating (significantly) younger people, as they felt that the generational differences would make them appear sexually unskilled or outmoded. They tended to equate younger generations with (more) sexual freedom and sexual experience, and to mark their own experiences as out of time. For example, one participant associated the excitement or the nervousness about new ways of having sex or having sex with people of the same gender with puberty. It shows that sexual experimentation in later life challenges heteronormative temporality and the life course. But it also shows how difficult it is for older people themselves to associate experimentation (and the insecurity associated with it) with later-life sexuality.

Participants who were with one or more younger partners pointed to some unpleasant consequences of disrupting heteronormative temporality. Annick, who was in her mid-fifties and had begun a sexual relationship with a younger woman a few years ago, explained that she enjoyed having sex with her new partner and experiencing new sexual pleasures, but that people around her referred to her new relationship as ‘a midlife crisis.’ Those reactions, which dismissed her new relationship as merely the result of her inability to accept the transition to older age, made her feel that her experience was not considered real. Castro Varela and Bayramoğlu (2021) note that irritation, anger, or astonishment are reactions that, despite society’s increasingly flexible expectations of the life course (e.g. it is no longer shameful to get divorced), indicate that some things are still denounced as having happened at the wrong time.

Vera, however, explained that dating younger men after her divorce had increased her sexual confidence. She clarified that being ‘in her sexual power’ was precisely what the younger men considered attractive about her. She said, ‘If I had been 20 years younger, I would really not have been able to understand why they would find [an older woman] so attractive. But for them, it was almost hypnotic.’ By constructing herself as a hypersexual being, she denounced the gendered and aged inequalities that desexualize older women. Similar to what Montemurro and Siefken (2014) observed in their study on the use of the term ‘cougar,’ Vera experienced being sexualized as a welcome alternative to narratives that deprive older women of possibilities to position themselves as sexual beings. Nevertheless, Vera continued that after some time spent on digital dating platforms and meeting people for sex, she grew tired of meeting people who were unwilling to commit or enter into an intimate relationship. She explained that she had given up on digital dating because the (predominantly heterosexual) men she met on these platforms were looking for superficial sex, cheating on their partner, or ‘trying out’ polyamory (but changed their minds once they had the opportunity to have a monogamous relationship with another woman). Vera regretted that she was unable to find the intimacy and connection that she was looking for when having sex with the people she met on these digital dating platforms. This illustrates that stereotypes of older women – even if they affirm that older women are sexual beings – are limiting and do not necessarily change older women’s position in the (hetero)sexual patriarchal order.

### Sex and affirmative old(er) age

Participants often described time as limited and often referred to time in the past as ‘wasted’ time. Vera described how she felt the need to ‘catch up’ after her divorce, because she felt like she had many unexplored sexual fantasies and less and less time. She said, ‘I really had this thought, like, what if you are on your death bed tomorrow and look back on your life? Oh, I would regret so much.’ She started digital dating in her early fifties and clearly told her sex partners what she wanted to try, which she explained was only possible for her because growing older had brought her confidence. About one of her first sex dates with a younger man, she said: ‘I compare it with a volcano that is about to burst. I had [multiple] orgasms that evening, an explosion of sexual energy.’ She described the sexual energy as something that had to get out, which depicts living out her sexual fantasies as something almost inevitable. However, she also mentioned that she was surprised: ‘[It] was for me a pivotal moment where I started to think: who am I really? Do I really know myself? Because I surprised myself so much, I never knew I had that in me.’ Although she had always thought of herself as a sexual person, her new sexual experiences appeared to her like a catalyst for a growing self-awareness. Vera’s reflections coincide with Audre Lorde’s (2007 [1984]: 57) view on the erotic, who writes, ‘Our erotic knowledge empowers us, becomes a lens through which we scrutinize all aspects of our existence, forcing us to evaluate those aspects honestly in terms of their relative meaning within our lives.’ For Vera, this meant that her ‘erotic knowledge’ helped her re-evaluate the ageist stereotypes and expectations of later-life sexuality imposed on her.

Jo, in her fifties, talked about time in a different way. She described it as ‘a shame’ that until a couple of years ago she had never considered the possibility of simply not ‘giving a fuck’ about what other people thought about her sex life and relationships. She described her shift to the queer sex and relationships later in her life – and thus disrupting heteronormative temporality – as a choice for herself and her own pleasure. However, she also feared that the changes in her body would prevent her from having joyful sex in the future. This aligns with the observation made by Crip theory scholar Alison Kafer (2013: 37) that the precarious prognosis of becoming disabled (or dying) often becomes a driving force for ‘erotic investment in the present.’ When the topic of sexual fantasies came up, Jo mentioned that growing older for her was accompanied by an ‘urge to do as many things as fast as possible.’ Such an erotic investment in the present showed up in other participants’ stories in their description of feeling like they had to ‘catch up’ and enjoy everything ‘twice as much’ as they got older. While Jo mentioned that bringing her sexual fantasies to life was a lot of fun, she also jokingly added that ‘you want to have something to look back on when you’re in that wheelchair,’ thus illustrating the anxiety around the changes that may occur in her body in the coming years.

For several participants, both younger and older than Jo, bodily impairments and late-onset disabilities that interfered with their sex life were a reality already. These participants described newfound fatigue, pain, or injuries in their bodies, but framed them as reasons and opportunities to approach sex differently later in life. Changes mentioned by participants included the use of sex toys and trying sex in different positions (e.g. sitting on a chair instead of lying down to avoid pain and aggravating injuries). For some participants it was important to find the right time to have sex (e.g. having sex during the day instead of at night or scheduling sex with a partner in advance). Inge, who was in her fifties, said that it had become more difficult for her to get sexually aroused spontaneously, which she connected to menopause. She explained that she and her partner had become more communicative about sex than they had been a few years ago. She said, ‘I mean, you can have spontaneous sex and intimate and sweet sex, but you can also just say ‘let’s have sex now.’ That is possible. [In] the end it all leads to the same thing, it’s not like you have better or worse sex.’ These stories not only suggest that trying out new things is not just reserved for sex earlier in life, but also that bodily changes associated with growing older can inspire new ways of having or wanting sex.

Giselle, a non-binary participant in their fifties, mentioned that they felt more and more ‘relaxed’ about sex as they grew older. A couple of years before the interview, Giselle’s children had moved out of the house and they had gone through a divorce. They described these events as important moments in their life in which they found opportunities to reinterpret their understanding of sexuality and let go of their previous strict idea of what sex is or can be. In a way, Giselle placed their own experiences within a queer time that fits Halberstam’s (2005) definition, namely a model of temporality that emerges once one leaves the temporal framework of reproduction and longevity. While no longer living in heteronormative temporality contributed to Giselle’s broadening understanding of sexuality, they mentioned that their changing body was another important driving force behind this queer trajectory. They explained that they felt less sexually aroused than earlier in their life, but that their newfound interest in various other pleasurable activities was reassuring them that sex would remain possible and, more importantly, pleasurable. ‘Even just stroking or cuddling or massage are, I believe, intimate and can be done with a sexual, sensual mind and intention,’ they said. Giselle’s experiences therefore illustrate how a decrease in sexual arousal and desire can redefine or broaden what sex and sexual pleasure are.

Changes in the body are central in Linn Sandberg’s (2013) work on affirmative old age. She argues that the body should not be understood as raw material, but as something that has agency and can shape subjectivity and sociality. Important in Sandberg’s definition of affirmative old age is how the body can be theorized in terms of difference (changing bodies, arousals, and desires) without understanding it as marked by decline. When asked how they felt about broadening their definition of sex and pleasure later in life, Giselle replied,

I could feel stupid for not knowing [about how broad the definition of sex can be], I only learned after the age of 50, so it was like, wow, I had a lot of years of ignorance and not knowing. It is a bit embarrassing, to be honest. But it’s also a feeling of relief […], a reduction of pressure and performance expectation, and so on.

Giselle explained that they had begun to include practices such as BDSM in their definition of sex and pleasure later in life, because the separation of giving and receiving pleasure increased their enjoyment of each position. In Giselle’s experience, BDSM allowed for sexual pleasure that is not necessarily linked to physical sexual arousal. As their sexual arousal had changed over the years, practicing BDSM was a welcome alternative to heteronormative, penetrative sex that requires high states of physical arousal for the sex to be pleasurable for both partners. Giselle’s motivation for including BDSM in their definition of sex therefore did not seem to align with a neoliberal, linear understanding of sexual liberation as ‘kinkier sex’ or ‘more sex.’ It was rather a way to navigate their decreasing sexual arousal later in life. Similarly, Giselle described non-monogamous relationship styles as potentially helpful in reducing the pressure of maintaining sex in relationships later in their life. They said, ‘you can be sexual or sensual with a low libido, and also if you’re ‘poly’, your partners don’t have to rely on you for all of their sexual pleasures.’ While Giselle’s experiences show that sexual arousal can indeed decrease when one gets older, their experiences show that this is not necessarily problematic. Their story is also disruptive of normative life course trajectories, as it were non-normative sexualities and relationships styles that played an important role in navigating their changing sexual desires and arousal later in life.

## Discussion and conclusion

By analyzing the stories of older queer women and non-binary people in Belgium and the Netherlands, this paper makes an empirical contribution to studies on life courses, temporality and (queer) sexuality. The participants’ stories varied and used different strategies to process their experiences. However, we found that the frame from which people drew their sexuality often continued to be based on a linear temporality. Their stories reinterpreted the past to reinforce ‘queer’ coherence, and in doing so they disturbed the linearity of heteronormative temporality. Queer coherence supposes consistency, but – as Kadji Amin (2014: 220) puts it – ‘at the expense of episodes or fleeting moments that would fracture or exceed it.’ While heteronormative expectations weighed heavily in the stories of the participants as a motivation to marry a man, their stories also revealed alternative layers in their motivation to adhere to heteronormative expectations at the time, including having children and compulsory sexuality.

Academic research has shown that the hegemonic discourse on sexuality desexualizes older women because of their age more so than men (Calasanti, 2005; Bouson, 2016). In the participants’ stories, awareness of sexageist assumptions and internalized ageism blended with resistance to ageist oppression, showing that there is room for reinterpretation and revaluation of sex later in life. The stories of participants that dated younger people add to the existing academic literature that focuses mostly on older women’s experiences with men of the same age (see Vandeweerd et al., 2016; Fileborn et al., 2015; Watson & Stelle, 2011). At times the sexualization of older age prevented participants from finding the intimacy and connection they sought, but having sex with younger people also proved pleasurable and even empowering. While the dominant discourse prevents women from exploring previously unexplored ways of having sex, doing so brought increased self-awareness, confidence, and reassurance. What enabled them to challenge the logics of heteronormative, linear time were not only the historical and societal circumstances. Growing older itself at times provided participants the necessary confidence to pursue their queer sexual desires and fantasies. Conversely, pursuing these desires and fantasies later in life was often named as contributing to increased sexual self-awareness and confidence.

Using a lens of queer temporality and affirmative old age allows us to challenge the dominant framing of sexual liberation in Western queer discourse, which is often associated with an understanding of sexuality that equates transgression with liberation. However, our findings show that understandings of sex can change and that relationship styles can evolve in non-linear ways. We have shown that sexuality in later life can be contradictory and fluid, and that some ways of relating to sex (having more or perhaps kinkier sex later in life) are no more liberating than others (e.g. choosing to stop having sex). Centralizing the usually marginalized experiences of older queer women and non-binary people in this paper has proved helpful in looking beyond prevailing phallocentric and orgasm-oriented assumptions of sex in the “good” later life. However, many of the findings in this paper may resonate with older people of any sexual or gender identity, as challenging the role and shape of sex in hegemonic heteronormative life courses may come in many different and unexpected ways.

Discussing the impact of physical changes shows how much the inclusion of older people in queer studies has to offer in terms of enhancing our understanding of how material and discursive aspects intertwine to transform the sexual self and people’s understanding of it. The fear of the future in the participants’ stories seemed to be based on prevailing consumerist and performance-oriented ideas that assume that “good sex” is less and less attainable over time. All participants experienced differences in their bodies, desire, and sex lives compared to their younger years. However, the affirmative view of older age that we adopt allows us to see the (older) body differently, not just as a body in decline, or a body that is able to remain “youthful,” but as a body that offers opportunities for new experiences that can help us rethink gender, embodiment, and sexuality. We argue that an affirmative account of sexuality in later life can form the basis for a politics of resistance to the sexageist stereotypes that pervade the sexuality of all women and non-binary people in later life.

Suggestions for further research include examining relationship preferences in later life. A focus on the ways in which older queer women and non-binary people deal with age differences between partners can make a valuable contribution to theorizing the way ageism operates within conceptualizations of queer temporality. However, the practices of older people in relation to non-monogamy also deserve further investigation, as their experiences have received little attention in polyamory research to date. Academic research on older women and sex has looked at the increased confidence and sense of sexual freedom (see Fileborn et al., 2015; Rowntree, 2014) and the subsequent refusal of older (heterosexual) women to repeat (hetero)normative relationship structures in the relationships they entered into later in life (e.g. they prefer to live apart from their (future) partner(s) (Watson & Stelle, 2011)), but what is missing is how this may lead older people to non-monogamy and how gendered dynamics structure their experiences.

## Data Availability

The participants of this study did not give written consent for their data to be shared publicly, so due to the sensitive nature of the research supporting data is not available.
